# Case report and literature review: Resection of retroinfundibular craniopharyngioma via endoscopic far-lateral supracerebellar infratentorial approach

**DOI:** 10.3389/fonc.2022.976737

**Published:** 2022-10-28

**Authors:** Yang Bai, Xiaoyu Sun, Xinning Li, Song Han, Guobiao Liang, Sizhe Feng, Chunyong Yu

**Affiliations:** Department of Neurosurgery, General Hospital of Northern Theater Command, Shenyang, Liaoning, China

**Keywords:** supracerebellar infratentorial approach, endoscope, surgical approach, retroinfundibular craniopharyngioma, case report

## Abstract

**Introduction:**

The management of retroinfundibular craniopharyngioma (CP) remains the ultimate challenge for both transsphenoidal and open transcranial surgery because of their anatomical location and proximity to vital neurovascular structures. In this report, we aim to describe the technique and feasibility of a novel approach, the purely endoscopic far-lateral supracerebellar infratentorial approach (EF-SCITA), for resection of retroinfundibular CP.

**Case description:**

A 63-year-old women presented with progressive visual disturbance, polyuria, and spiritlessness of a 3-month duration. Imaging studies revealed a typical retroinfundibular CP containing solid and cystic components with calcification, which extended inferiorly in front of the brainstem and upward into the third ventricle. The EF-SCITA approach was attempted for resection of the tumor. During surgery, lateral prone positioning with upper flexion of the head and early CSF release allowed for download retraction of the cerebellum. This, in combination with tentorium incision, created a working corridor toward retrosellar and suprasellar spaces. This approach required working between neurovascular structures in the crural cistern, with tumor removal permitted in supra-oculomotor and infra-oculomotor spaces. After aspiration of the fluid contents through the supra-oculomotor triangle, the solid lesion was found tightly adhering to the distal part of the pituitary stalk, and subtotal resection was achieved for maintaining the integrity of pituitary function. In the immediate postoperative period, the patients exhibited oculomotor paralysis and was discharged with hormonal replacement therapy three weeks after operation. At her three-month follow-up appointment, she reported obvious vision improvement. Physical examinations showed partial alleviation of oculomotor paralysis. Pathological analyses confirmed the diagnosis of papillary CP.

**Discussion:**

The purely EF-SCITA approach combines the advantages of both the posterolateral approach and endoscopic technique, which offers access to retrosellar and suprasellar spaces with seemingly low risks of postoperative morbidity. It would be a safe and effective alternative for the treatment of retroinfundibular CP, especially those with lateral extension to the temporal lobe or posterolateral extension to the petroclival region. Further observational studies in a larger cohort are urgently needed to assess the long-term efficacy of this minimal access approach.

## Introduction

Craniopharyngioma (CP) is a rare epithelial tumor occupying the midline sellar/suprasellar space, accounting for 1.2%-4.0% of all intracranial tumors (1). Despite its benign appearance, it can be associated with unfavorable prognosis due to its proximity to critical neurovascular structures, particularly the visual pathway and hypothalamus. Therefore, surgical removal remains the first-line therapy and offers the best chance of cure (2). Understanding surgical anatomy is essential for maximal tumor removal with minimal postoperative morbidity. The origin, growth pattern, and relationship of CP with neighboring vital tissues is pivotal for choosing the best approach for surgical exposure in weighing risks of aggressive resection against hypothalamic function impairment and tumor recurrence (3)

Several classifications have been introduced to aid in surgical planning. Based on relative location to the optic chiasm, CP was originally described as intrasellar, prechiasmatic, subchiasmatic, retrochiasmatic, and purely intraventricular (4). According to the pattern of suprasellar extension, CP could also be classified as preinfundibular, transinfundibular, and retroinfundibular, which would be equivalent to the prechiasmatic, subchiasmatic, and retrochiasmatic type, respectively (5, 6). There is consensus on transcallosal/transcortical approach for the treatment of intraventricular tumors and transsphenoidal surgery (TS) for intrasellar lesions. However, the optimal approach for other suprasellar subtypes still remains a matter of debate (6). Among them, the retrochiasmatic-retroinfundibular subtype, comprising 11-46% of all CPs, is the most intricate to treat given its hidden position behind the pituitary stalk, upward extension into the third ventricle (TV) and downward extension into the interpeduncular cistern and retrosellar region (7). After years of surgical explorations, several approaches (including anterior midline approaches, anterolateral approaches, and the petrosal approach), microscopically or endoscopically, have been developed to reach retroinfundibular CPs. However, the exposure for retroinfundibular CPs is restricted with these conventional approaches compared with the exposure of other subtypes (2, 7–10).

The supracrebellar infratentorial (SCITA) approach is first proposed in 1910 by Victor Horsley, with several variants making use of off-midline approaches established for accessing pathologies located from the pineal region to the petrous ridge (11, 12). As an important variant of the SCITA approach, the far-lateral SCITA approach is introduced for resection of lesions in the posterolateral tentorial gap, such as cavernous malformation, aneurysm, and glioma (13–16). Endoscopic neurosurgery is gaining momentum in the last decades, with the nasal cavity and infratentorial space established as ideal operating areas (17, 18). Recently, Xie et al. firstly used pure endoscopy in the SCITA space to resect petroclival meningiomas extending into supratentorial areas (12). Their successful experience with this approach motivated us to use the same technique in treating retrochiasmatic CPs. Herein, we report according to the CARE guidelines a case of the endoscopic far-lateral SCITA (EF-SCITA) approach in subtotal removal (STR) of retrochiasmatic CP, with specific emphasis on technical surgical nuances. In the discussion, we summarize breakthroughs and insights on operative approaches for the treatment of retrochiasmatic CP, and analyze the operation essentials, and strengths and weaknesses of each passage from the perspective of surgery.

## Case description

**Figure 1:**
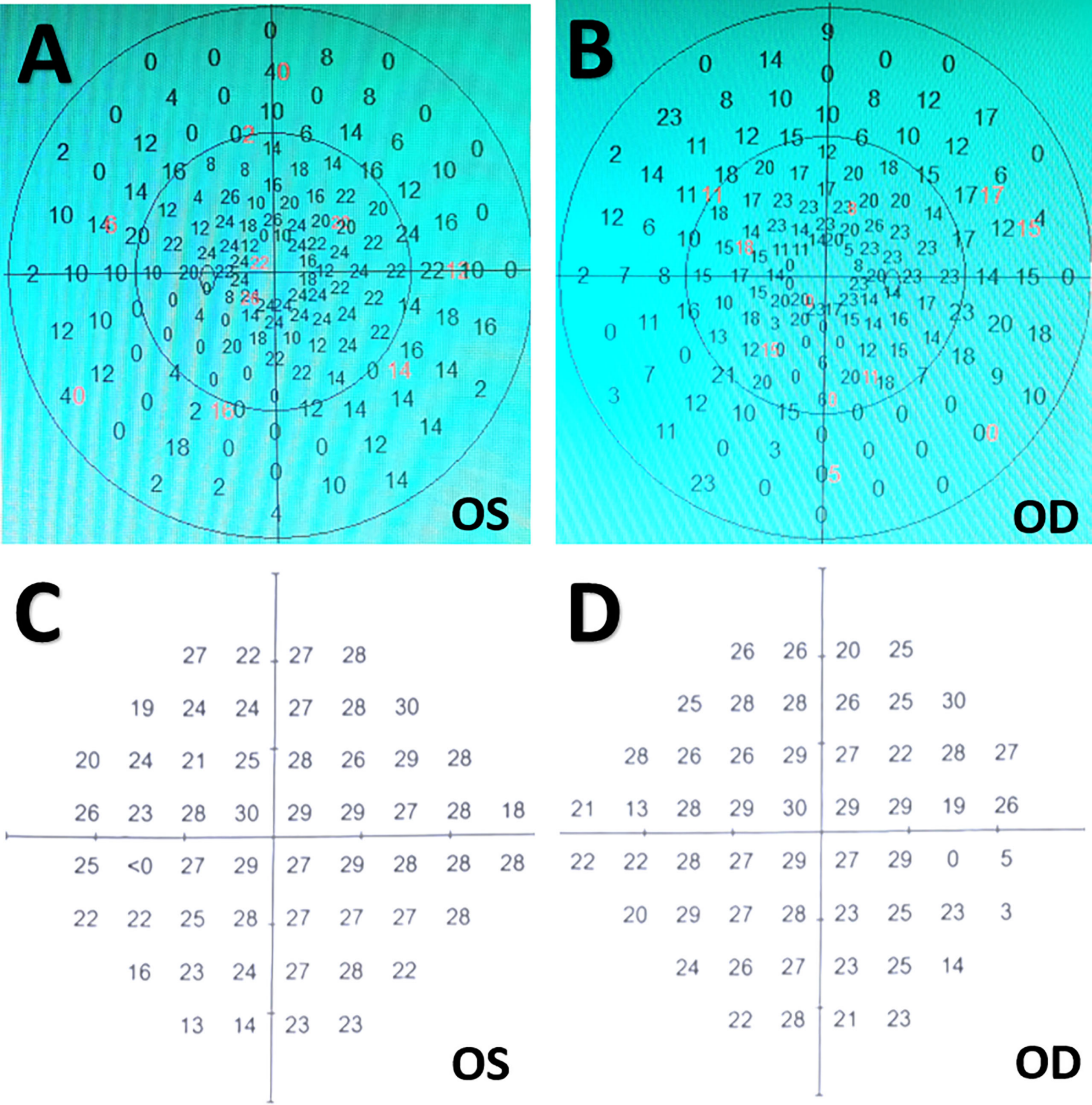
Visual field tests before and after surgery. **(A, B)** Preoperative ophthalmologic examinations indicating bilateral tunnel visual fields. **(C, D)** Postoperative visual field tests three months after surgery showing slight peripheral visual field loss in bilateral eyes. OD, oculus dexter; OS, oculus sinister.

A 63-year-old women presented with progressive visual disturbance, polyuria, and spiritlessness of a 3-month duration. Ophthalmological examination identified visual acuity in the right eye of 0.12 and in the left eye of 0.4, with bilateral tubular visual fields (**Figures 1A, B**). Routine laboratory investigations were normal. Endocrinological studies indicated increased serum levels of prolactin (48.02 ng/ml; ref: 2.74-19.64 ng/ml). Urinalysis showed low specific gravity of morning urine (1.005; ref: >1.020). No specific medical, family and psycho-social history was reported.

**Figure 2:**
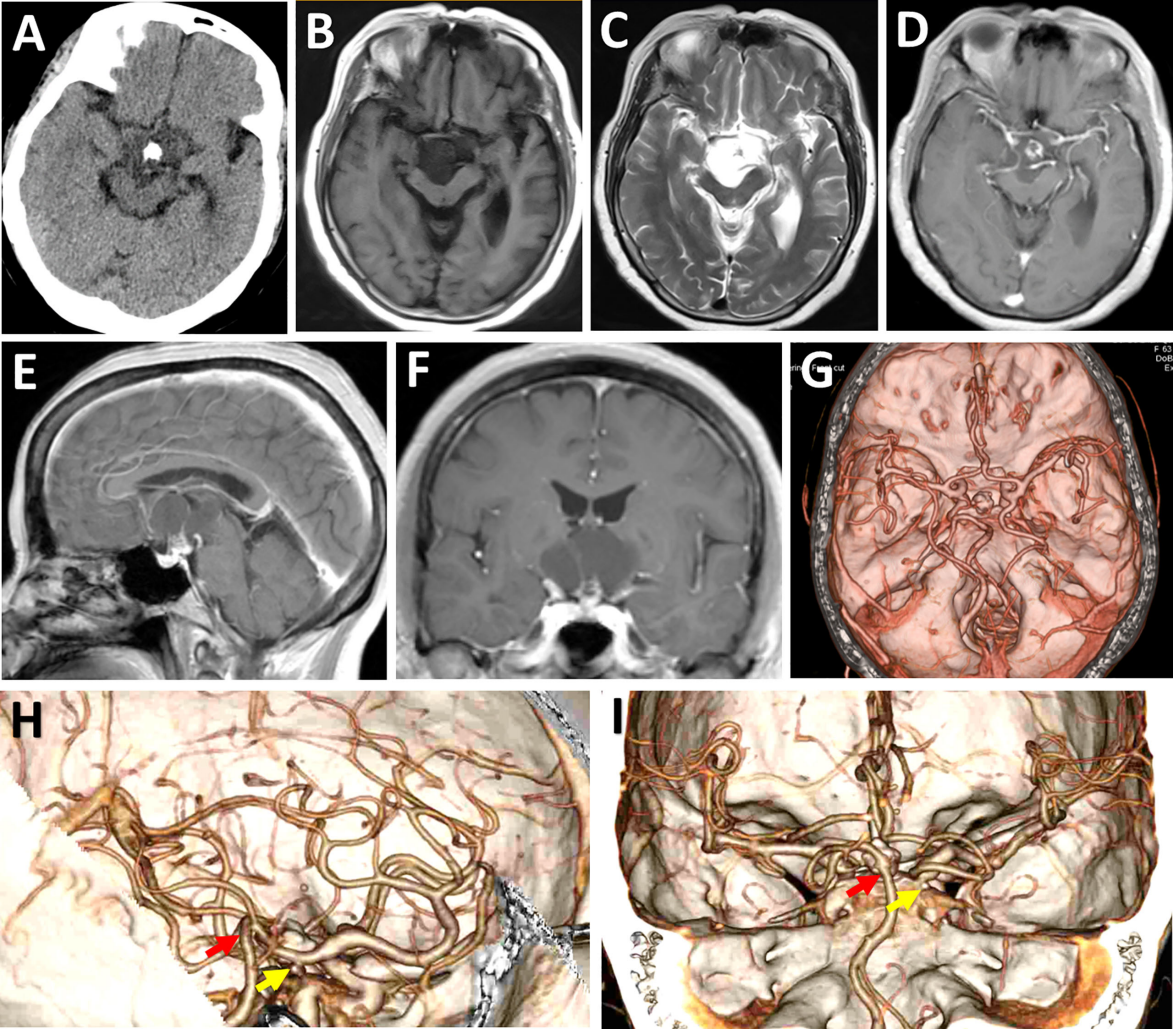
Radiological evaluation of the suprasellar lesion preoperatively. **(A-F)** Preoperative CT and MRI scans showing a typical retroinfundibular CP. This lesion manifested as a hypodense irregular mass with calcification located in the suprasellar lesion **(A)**, which was hypo-intense on T1WI **(B)** and hyper-intense on T2WI **(C)**. Post-contrast (gadolinium-enhanced) axial **(D)**, sagittal **(E)**, and coronal **(F)** MRI showed a thick, irregular enhancement of the solid part and a slight rim enhancement of the cystic component. **(G-I)** Preoperative axial **(G)**, sagittal **(H)**, and coronal **(I)** CT angiography images showed the relative location of the tumor to the Willis’ circle. Note that in this patient the basilar apex was at a higher position surpassing the posterior clinoid process. The yellow arrows denote posterior clinoid process, while the red arrows depict the basilar apex.

Computed tomography (CT) and magnetic resonance (MR) imaging revealed a well-circumscribed, multilobular, suprasellar mass containing solid and cystic components with calcification (**Figures 2A-D**). This lesion was found to occupy the retro-infundibular space and extend inferiorly in front of the brainstem and upward into the TV, displacing the midbrain posteriorly and the optic chiasm anteriorly (**Figure 2E**). Gadolinium-enhanced MR imaging demonstrated a thick, irregular enhancement of the solid part and a slight rim enhancement of the cystic component (**Figures 2D-F**). The mass measured 3.1 × 4.0 × 2.1 cm in the sagittal, axial, and coronal planes, respectively. The pituitary stalk was clearly shown in the midline on the coronal section (**Figure 2E**). CT angiography (CTA) revealed the existence of right fetal posterior communicating artery (PComA) (**Figure 2G**) as well as the relative location of the tumor to the Willis’ circle (**Figures 2G-I**). Based on these clinico-radiological features, CP was proposed as the most likely diagnosis, and the EF-SCITA approach was attempted for tumor resection. The timeline of the overall therapeutic process is shown as a flow diagram in **Supplemental Figure 1**.

**Figure 3:**
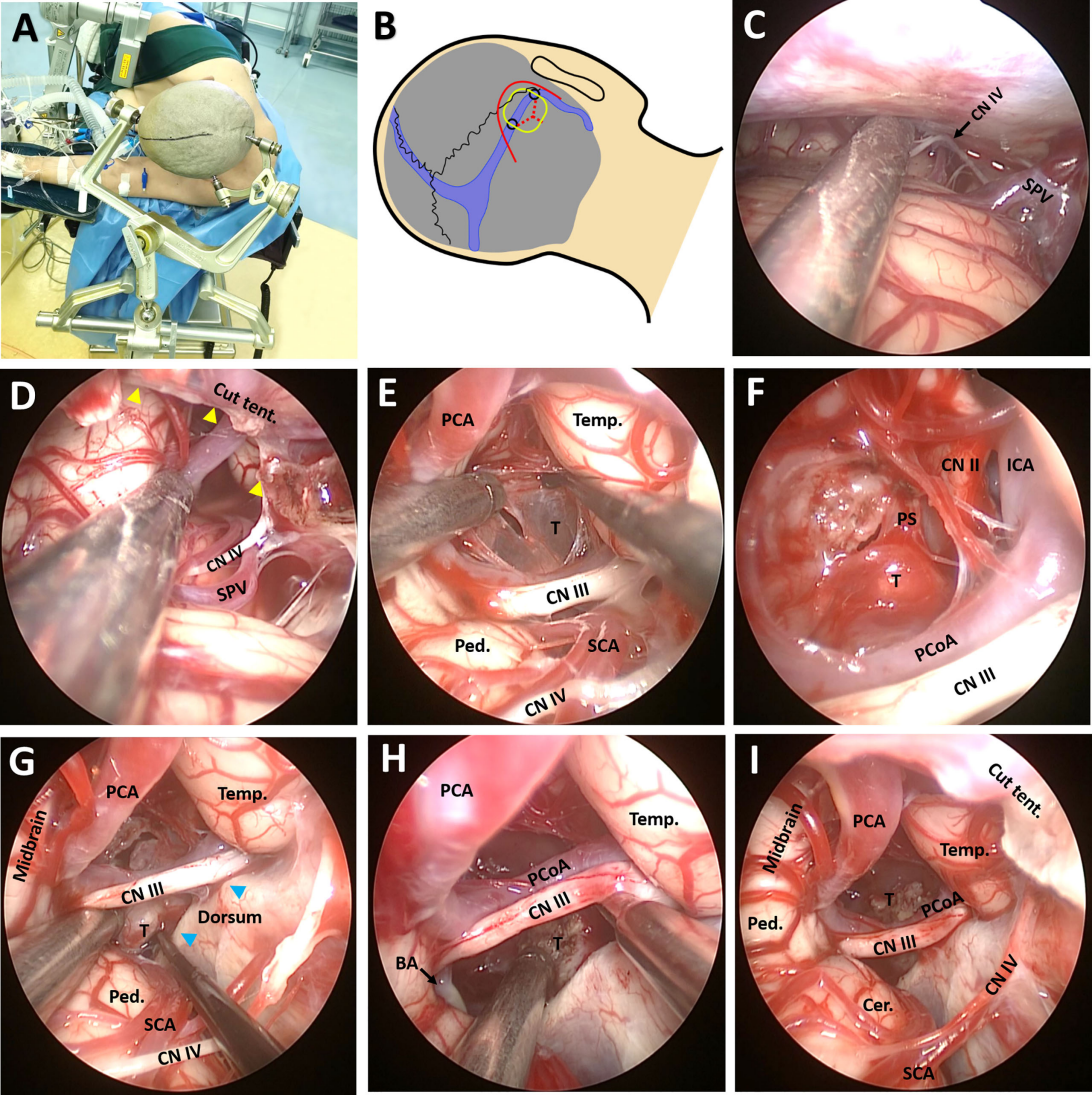
Surgical procedure and nuances of the EF-SCITA approach. **(A)** The lateral prone position for a right-sided EF-SCITA approach. **(B)** Head position and surface landmarks for the transverse/sigmoid, the curved skin incision (red line), the proposed bone window (yellow rounded rectangle), the mark for drilling sites including the asterion and another site located 3.5 cm medial to the asterion (black circles), and the Y-shaped durotomy (red dotted lines). **(C)** Endoscopic explorations in the lateral supracerebellar infratentorial space. **(D)** The incision of the tenrorium for exposure of the crural cistern. The yellow arrowheads denote the edge of the cut tentorium. **(E)** The exposure of the posterior capsule of the cystic lesion in the supra-oculomotor space. **(F)** The exposure of the solid part of the tumor in the supra-oculomotor space after aspiration of the contents. **(G, H)** Dissection of the solid tumor from the pituitary stalk in the infra-oculomotor space. The anatomical boundaries of the supra-oculomotor and infra-oculomotor spaces are clearly seen. The blue arrowheads indicate bilateral posterior clinoid processes. **(I)** The endoscopic view of subtotal removal of the tumor, with a small part of the solid lesion adhering to the pituitary stalk left. BA, basilar artery; Cer, cerebellum; CN II, optic nerve; CN III, oculomotor nerve; CN IV, trochlear nerve; ICA, internal carotid artery; PCA, posterior cerebral artery; PCoA, posterior communicating artery; Ped., pedunculus cerebri; PS, pituitary stalk; SCA, superior cerebellar artery; SPV, superior petrosal vein; T, tumor. Temp., temporal lobe; Tent., tentorium.

The patient was placed in left lateral position with the right side on top. The head was fixed with a Mayfield clamp and placed in upper flexion of 30° to allow gravity retraction of the cerebellum (**Figures 3A, B**) (12). The endoscope monitor (Karl Storz GmbH and Co., Tuttlingen, Germany) was placed in front of the patient, while the endoscopic pneumatic arm holder was placed on the contralateral bedside. During surgery, the endoscope (0°, Hopkins II, Karl Storz GmbH and Co.) was fixed to the holding arm, thus allowing two-hand operation. A C-shaped retroauricular incision, resembling that seen in the retrosigmoid sinus approach, was performed to expose the suboccipital region and the mastoid bone. Then, the transverse sinus and the inner edge of the sigmoid sinus were revealed through suboccipital craniotomy (3.5 cm × 3.5 cm). A Y-shaped duratomy was then made, with the superior part of the dural flap turned over to the margin of the transverse sinus (**Figure 3B**). Gravity retraction, together with intraoperative cerebrospinal fluid draining (approximately 40 ml) with the aid of a preoperatively inserted lumbar drainage tube, expanded the infratentorial space allowing endoscopic explorations in the lateral superior cerebellar space and exposure of the superior petrosal vein (SPV), trochlear nerve (CN IV), and tentorium (**Figure 3C**).

A 1-cm incision was made in the anterior part of the tentorium for exposing the crural cistern (**Figure 3D**), with much attention paid to the CN IV during the procedure. After careful dissection of the CN IV and superior cerebellar artery (SCA), the oculomotor nerve (CN III) and posterior communicating artery (PCoA) could be identified. Under direct view, the crural cistern was separated into two spaces by the CN III: the supra-oculomotor triangle formed by the midbrain, temporal lobe, and CN III, as well as the infra-oculomotor triangle formed by the cerebral peduncle, dorsum sellae, and CN III (**Figures 3E-G**). After dissection of the supra-oculomotor space, the posterior portion of the cystic lesion was revealed (**Figure 3E**) and “motor-oil”-colored fluid could be drained from the cyst. After opening the capsule, the solid lesion in front of the pituitary stalk (PS) could be easily identified in the suprasellar cistern, together with right internal carotid artery (ICA) as well as the displaced optic nerve (**Figure 3F**). The basilar artery could also be observed in the infra-oculomotor space when uplifting the CN III and PCoA (**Figure 3G**). Then, the solid part was removed in a piecemeal fashion in the infra-oculomotor space, except for the calcified part blending into the distal part of the PS (**Figures 3G-I**). Finally, the capsule was bluntly removed from the surrounding reactive gliotic layer. Of note, the PS, cranial nerves, and vessels should be critically monitored when manipulating this region.

**Figure 4:**
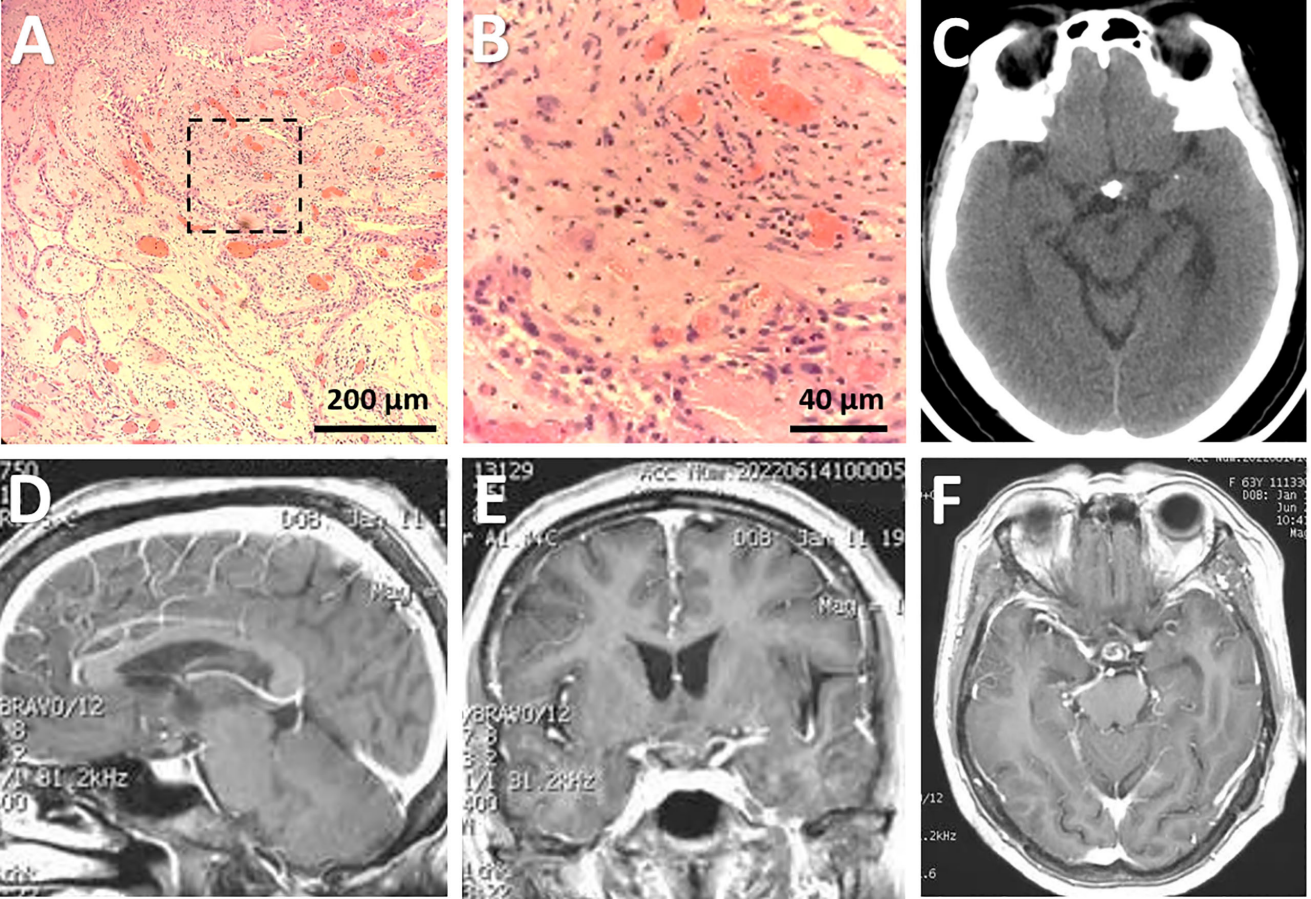
Pathological and radiological evaluation of the lesion postoperatively. **(A, B)** Photomicrographs of hematoxylin-eosin staining showing typical features of papillary craniopharyngioma. The framed area in **(A)** was magnified in **(B)**. Bars = 200 μm in **(A)** and 40 μm in **(B)**. **(C-F)** Postoperative CT **(C)** performed immediately after surgery and post-contrast sagittal **(D)**, coronal **(E)**, and axial **(F)** MRI scans performed three months after surgery showing subtotal removal of the lesion, with a few calcified components left in the suprasellar region.

In the H&E staining, the squamous cell epithelium of the tumor was not easily recognizable (**Figures 4A, B**). However, in subsequent immunohistochemical investigations, the morphological characteristics of this type of epithelium were evident, particularly in the CK 5/6 staining (**Supplemental Figure 2**). The underlying stroma was edematous and fibrotic with several congested vascular channels (**Figures 4A, B**). About five percent of tumor cells expressed Ki67 (**Supplemental Figure 2**). Further molecular pathologic analyses showed that the tumor exhibited *BRAF* (V600E) mutations instead of *CTNNB1* mutations. Thus, the final diagnose of papillary CP was confirmed.

Postoperative imaging examinations demonstrated STR of the tumor (**Figures 4C-F**). In the immediate postoperative period, the patient exhibited exotropia, hypotropia, and complete ptosis of the upper lid in the right eye. The diabetes insipidus subsided one-week after surgery with vasopressin treatment. Three weeks after operation, she was discharged with hormonal replacement therapy, requiring the same dose of hormones as prescribed preoperatively. At her three-month follow-up appointment, she reported complete alleviation of ptosis of the upper lid but still had extraocular muscle paralysis. Ophthalmological examination showed her visual acuity improved to 0.8 in the left eye and 0.5 in the right eye, and bilateral vision fields has almost returned to normal except for slight peripheral defects (**Figures 1C, D**). A longer-term follow-up is warranted for monitoring oculomotor functions. Since the patient refused gene targeting therapy, irradiation was suggested for reducing tumor recurrence.

## Discussion

**Figure 5:**
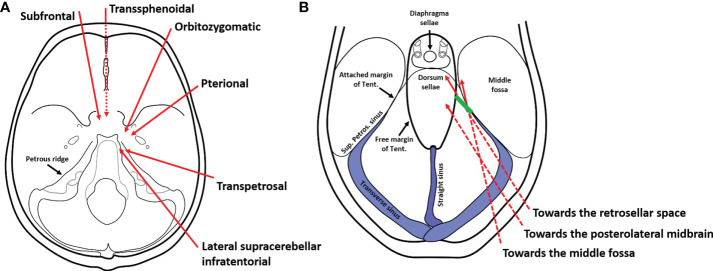
Schematic diagrams of surgical approaches for the resection of retroinfundibular craniopharyngiomas. **(A)** Current approaches for the resection of retroinfundibular craniopharyngiomas include anterior midline approaches (the transsphenoidal approach and the subrontal approach), anterolateral approaches (the peterional approach and orbitozygomatic approach), and posterolateral appoaches (the transpetrosal approach and the lateral supracerebellar/infratentorial approach described herein). The dotted line indicates the surgical corridor underneath the skull base. **(B)** The lateral supracerebellar/infratentorial approach was originaly used for resection of lesions in the posterolateral midbrain and tentorial region. However, the incision of the tentorium broadens the range of exposure towards the retrosellar space for resection of retroinfundibular craniopharyngiomas as well as the middle fossa for resection of petroclival region meningioma with supratentorial extenstion. The dotted line indicates the surgical corridor underneath the tentorium. The green line denotes the incision site of the tentorium.

In this report, we presented a case of a women harboring typical retrochiasmatic-retroinfundibular CP. This tumor is bounded laterally by the CN III, PCoA, and perforating branches (including the tuberoinfundibular artery), posteriorly by the basilar apex, PCA, posterior perforated substance, and crus cerebrii, anteriorly by the PS and dorsum sellae, superiorly by the infra and retrochiasmatic surface including mamillary bodies, infundibulum, anteromedial hypothalamus, and TV, and inferiorly by the prepontine cistern (6). Traditionally, different anterior midline and lateral approaches have been used to approach retroinfundibular CPs, with their inherent limitations illustrated in previous studies (2, 6). Herein, we tested the feasibility of a novel posterolateral corridor, the EF-SCITA approach, in the removal of this kind of tumor with complex anatomic boundaries. Next, we would summary reported approaches for retroinfundibular CPs (**Figure 5A**), with their rationale, merits, and disadvantages elaborated.

Endoscopic endonasal TS (EETS) represents a paradigmatic shift in the neurosurgical approach to suprasellar lesions including CP (17, 19, 20). EETS provides direct visualization of infrachiasmatic area as well as the TV and hypothalamus without brain retraction, which is usually a blind pot from a transcranial view (6, 21, 22). Preinfundibular CPs are effectively resected through extended transtuberculum/transplanum TS; transinfundibular lesions require the addition of a transsellar approach with inferior pituitary transposition; and retroinfundibular CPs are better accessed by performing complete superior clivectomy with superior pituitary transposition (6, 23, 24). There are several limitations in this approach. Firstly, the major criticism is a greater incidence of CSF leak even with meticulous skull base reconstruction (17). Secondly, the resection of retroinfundibular CPs still remains challenging transnasally since careful microdissection of vital neurovascular structures that the tumor are densely adherent to can be difficult or even dangerous. Thirdly, EETS is unsuitable for patients with large-sized CPs, CPs with obvious lateral extension, and non-peumatized sphenoid sinus (2, 25, 26). Fourthly, pituitary transposition may impair the arterial supply and venous drainage of the gland, leading to endocrine hypofunction (6, 24). Finally, it necessitates a steep learning curve with two surgeons skilled in skull base and endoscopic techniques (27).

With the shortest route to the suprasellar space, the pterional approach is suitable for most CP subtypes including those locating in or on the saddle, retroinfundibular CPs, and those extending into the TV, interpeduncular fossa, or the Sylvian fissure (9, 10). Multiple natural spaces including the optico-carotid and carotid-oculomotor corridors facilitate tumor resection without significant frontal lobe retraction (28), while the operating space gained by laminar terminalis resection further allows CP removal from the lower and anterior TV (29). Notably, orbitozygomatic modification of pterional craniotomy further facilitates the exposure of the interpeduncular cistern and improves maneuverability of surgical instruments (2), and the temporal or subfrontal extension of this approach gains wider tumor exposure for accommodating to the complicated invasive feature of CP (28). However, anterolateral approaches has certain drawbacks. Firstly, the angle of attack to the optic chiasm and lamina terminalis is oblique, which prohibits visualization of the ipsilateral wall of the TV and hypothalamus and thus conveys higher risks when blindly removing adherent tumor from the hypothalamus (7). Secondly, the lateral view through the natural corridors en route to the interpeduncular cistern and infrachiasmatic region is narrow, and is sometimes obstructed by ipsilateral perforating arteries from the PCoA and ICA (8). Thirdly, the superior viewing angle to the top of the TV is also insufficiently visualized by this approach, although it could be improved with an orbitozygomatic approach to some extent (7). Last but not least, the pterional approach affords poor visualization of contralateral opticocarotid triangle and retrocarotid space (30).

The transbasal subfrontal approach is another anterior midline approach proven advantageous when resecting giant retrochiasmatic CPs. Compared with anterolateral approaches, the subfrontal approach renders straight frontal trajectory to the anterior TV by maximizing the lamina terminalis working corridor, which provides the significant advantage of visualization of both walls of the TV and hypothalamus (7). Retroinfundibular CPs can also be followed inferiorly into the interpeduncular cistern through the lamina terminalis exposure (7, 31). Bilateral median approaches are also superior to unilateral ones in offering a wider working window with bilateral views of vital neural structures and perforating arteries (32). Inherent limitations of this craniotomy include the risks of potential violation of the frontal sinus and sacrifice of the ACoA and CN I. In addition, draining veins might be cut from the frontal lobe to the sagittal sinus. This maneuver, combined with prolonged retraction, carries higher risks of postoperative cerebral ischemia and contusional hemorrhage (33).

The posterior petrosal approach (or pre-sigmoid approach) was firstly advocated by Hakuba et al. for the treatment of large retrochiasmatic CPs. It necessitates manipulation of the contents in the crural cistern (CN III, CN IV, PCoA and perforating branches, and the second segment of PCA) to access the interpeduncular space (34). This transpetrosal-transtentorial approach provides a forward and upward oriented corridor allowing for dissecting the upper portion of the tumor which projects high into the TV. Using this approach, the surgeon can achieve direct visualization of the inferior surface of the optic chiasm, hypothalamus, and PS, while maintaining blood supply to these structures and preserving their function integrity (8, 34). However, this approach carries significant risk for temporal lobe contusion and venous issues (damage to the sigmoid sinus, petrosal vein, the Vein of Labbe, etc), renders operational complexity of mastoidectomy in young children with small and poorly aerated mastoid sinus (31), and also has the aforementioned shortcomings associated with lateral craniotomy.

To the best of our knowledge, this is the first case report describing the EF-SCITA approach in the treatment of CP. Several positions have been used for the EF-SCITA approach. The sitting or semi-sitting position could avoid cerebellar retraction-related injuries, but has risks of air embolism (13, 35). In this case, the lateral position was adopted, with upper flexion of the head allowing for download retraction of the cerebellum. The supracerebellar space was further enlarged with the aid of preoperative CSF release *via* lumbar catheterization. This surgical position, in combination with tentorium incision (**Figure 5B**) creates a working space for approaching retroinfundibular CP. This posterolateral approach requires working between neurovascular structures in the crural cistern, with tumor removal permitted in supra-oculomotor and infra-oculomotor spaces.

An interesting issue in this case is the existence of fetal PComA on the right side. Comparing to fetal PComA, normal PComA is usually thinner and shorter, and has perforating branches. Thus, normal PComA could instead restrict the surgical space and be more susceptible to operative injury (36–38). Considering this, we selected the side with fetal PComA for surgery. Surprisingly, we found that the fetal PComA, running behind and in parallel with the CN III, did not complicate the surgery corridor, and was not injured during surgery partly owing to the protection of CN III. However, the continuous PCA running along the edge of the midbrain to some degree limited the operative exposure of the approach.

An important reason why we did not choose anterior midline approaches was that manipulation of the tumor from the anteroposterior direction may cause injury to the basilar artery since the basilar apex, at a high position surpassing the posterior clinoid process, possessed a close relationship with the posterior portion of the tumor in this patient (**Figures 2H, I**). However, under the condition of the posterolateral approach, the BA hidden beneath the midbrain in the infra-oculomotor triangle could be well-preserved. Considering these, it is important to consider the impact of the variations of the Willis’ circle as well as posterior circulation vessels when planning the EF-SCITA approach.

The EF-SCITA approach allows an excellent view into retrosellar and suprasellar spaces and makes removal of the lesion feasible. After the drainage of cyst, the related position of the solid tumor and PS could be identified under direct view. One major limitation of this case is that we achieved STR for the sake of maintaining the integrity of pituitary function. Thus, despite an appropriate surgical corridor, the degree of adhesion to adjacent structures also influences the attempt to achieve surgical radicality. In light of the anatomical panorama rendered by endoscopy, we now prefer the EF-SCITA approach in patients with retroinfundibular CP, especially those with lateral extension to the temporal lobe or posterolateral extension to the petroclival region. It might also be possible for the resection of intrasellar CPs with the advent of abrasion technique of the dorsum under endoscopy in the future. However, it may not be applicable for preinfundibular CPs owing to the obstruction by the PS and large intraventricular CPs due to the limited upward view. Whether this approach is suitable for the exposure of transinfundubular CPs merits further clinical practice.

There are several advantages for this approach. Firstly, in the suprasellar region occupied with various neurovascular structures, manipulations with these vital tissues are inevitable irrespective of the surgical corridor used. However, high-definition wide-angle visualization and close-up observation with endoscopy could minimize injury especially to perforators supplying the hypothalamus and pituitary gland. Secondly, this approach simplifies craniotomy procedures, with a small postauricular incision that does not affects the appearance of patients. Thirdly, in our EF-SCITA experience, using a pneumatic holding arm to fix the endoscope allows for bimanual surgical techniques. Compared with the assistant holding the endoscope under the condition of EETS, this mode improves working efficiency and reduces the injury caused by desynchronization between the operator and assistant.

Despite these merits, we are clearly aware that it is not without flaws. The biggest disadvantage is working through the cranial nerve plane, especially above and below the fragile CNIII, and augmenting the risk of post-operative cranial nerve palsy, as reflected by postoperative CNIII paralysis in this case. In addition, this approach may confer risks of damage to cerebellum and brainstem before access to the cisterns even with spinal drainage, as well as the cerebrovenous system (including the petrosal venous, transverse sinus, and sigmoid sinus). Moreover, this approach still could not avoid inherent shortcomings of traditional craniotomy such as CSF leakage and intracranial infection. Other inherent shortcomings of endoscopy included difficulties when handling deep bleeding and a steep learning curve.

In conclusion, the EF-SCITA approach combines the advantages of both posterolateral approach and endoscopic technique, which might be an effective and safe alternative for the treatment of the challenging retrofundibular CPs. Observational studies in a larger cohort are urgently needed to confirm the efficacy.

## Data availability statement

The raw data supporting the conclusions of this article will be made available by the authors, without undue reservation.

## Ethics statement

Written informed consent was obtained from the individual(s) for the publication of any potentially identifiable images or data included in this article.

## Author contributions

SF, XS, YB and XL have been involved in the operation and management of the patient. SF and CY designed the report. YB and XS reviewed the literature, drafted the article and prepared the figures. SH provided important academic inputs during surgery as well as during the revision of this manuscript. All authors contributed in editing of the manuscript and approved its final version.

## Funding

This research was supported by the National Natural Science Foundation of China (grant No. 82101318 to YB) and Science and Technology Plan of Liaoning Province (grant No. 2021JH2/10300116 to SF).

## Acknowledgments

We thank Dr XL in the Department of Pathology for her help in neuropathology analysis.

## Conflict of interest

The authors declare that the research was conducted in the absence of any commercial or financial relationships that could be construed as a potential conflict of interest.

## Publisher’s note

All claims expressed in this article are solely those of the authors and do not necessarily represent those of their affiliated organizations, or those of the publisher, the editors and the reviewers. Any product that may be evaluated in this article, or claim that may be made by its manufacturer, is not guaranteed or endorsed by the publisher.

## Supplementary material

Supplementary Figure 1 A flow diagram shows the overall therapeutic process. CT, computed tomography; DI, diabetes insipidus; MRI, magnetic resonance imaging.

Supplementary Figure 2 Immunostaining analysis revealed the expression of CK5/6 **(A)**, P63 **(B)**, P40 **(C)**, CD34 **(D)**, vimentin **(E)**, and Ki67 **(F)** in the tumor. Bar = 500 μm in **(A–F)**.
