# Supplementary figures and images for: Case report and literature review: Resection of retroinfundibular craniopharyngioma via endoscopic far-lateral supracerebellar infratentorial approach
Source: Front Oncol. 2022 Oct 28;12:976737. doi: 10.3389/fonc.2022.976737 (PMC9650989; doi:10.3389/fonc.2022.976737)

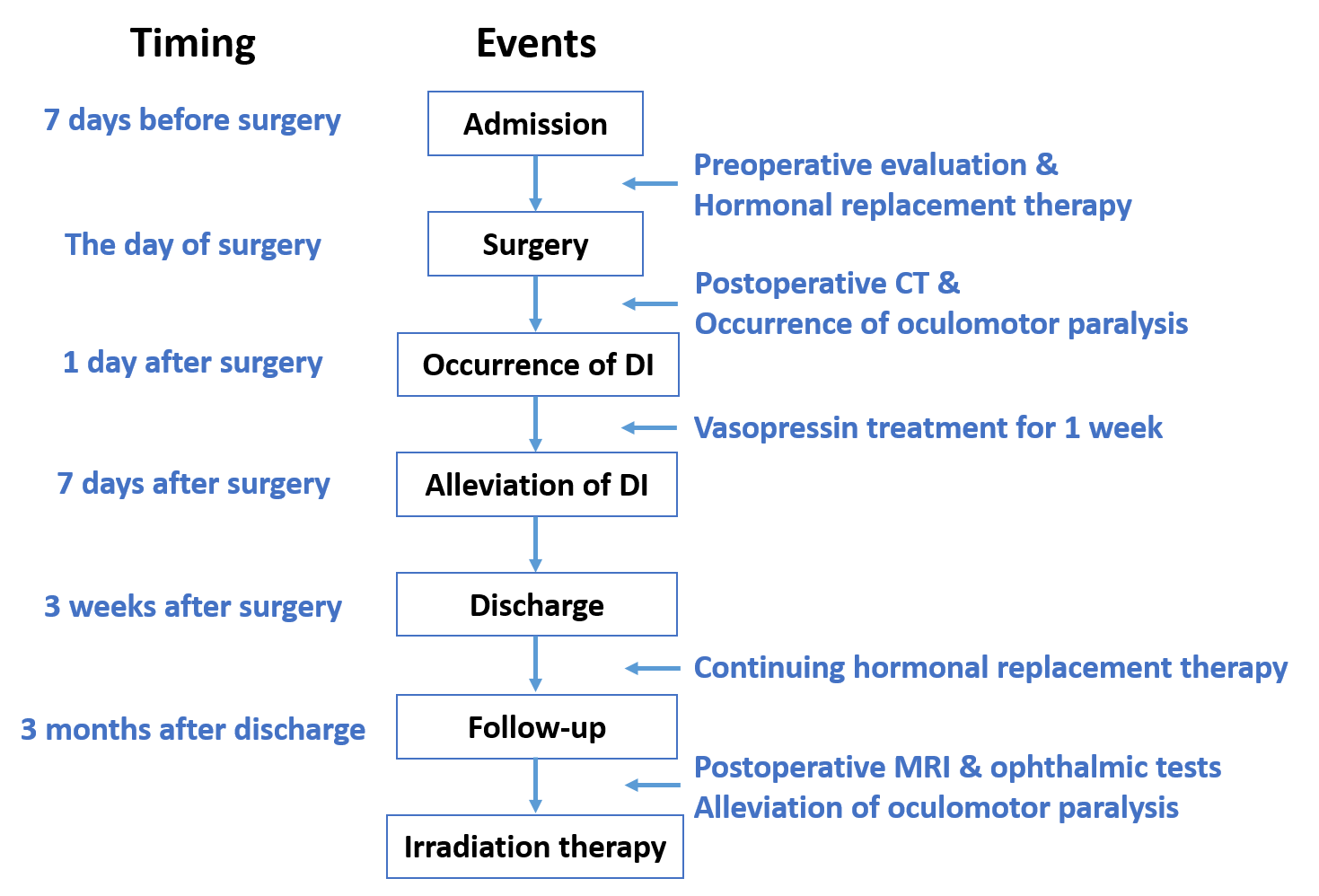

Supplement: Supplementary Figure 1 — A flow diagram shows the overall therapeutic process. CT, computed tomography; DI, diabetes insipidus; MRI, magnetic resonance imaging. [file Image_1.tif]

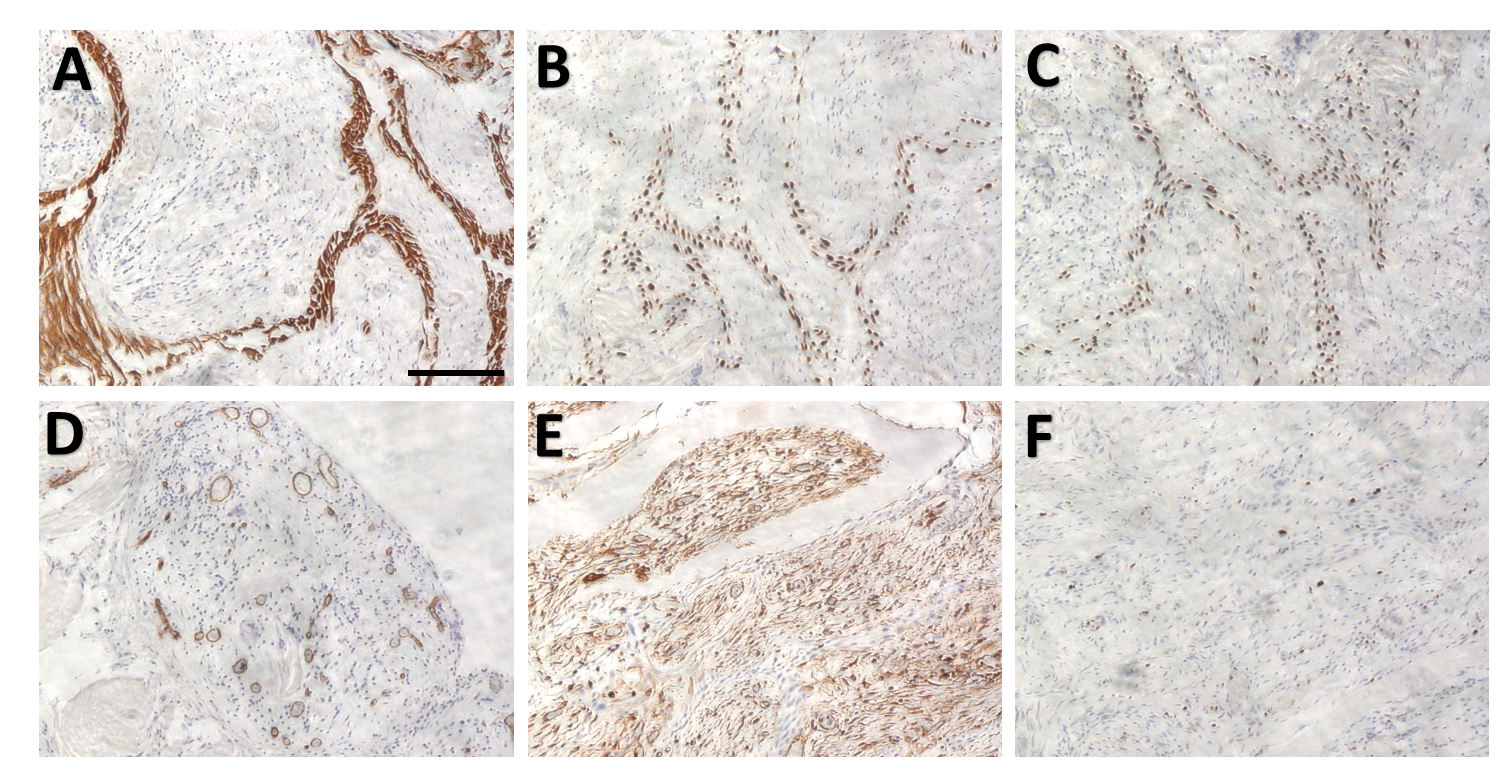

Supplement: Supplementary Figure 2 — Immunostaining analysis revealed the expression of CK5/6 (A), P63 (B), P40 (C), CD34 (D), vimentin (E), and Ki67 (F) in the tumor. Bar = 500 μm in (A–F). [file Image_2.tif]
